# Supplementary material for: Dynamics of the Lipidome in a Colon Simulator
Source: Metabolites. 2023 Feb 27;13(3):355. doi: 10.3390/metabo13030355 (PMC10051596; doi:10.3390/metabo13030355)

Supplementary Figure $S1$: SEA in four vessels (V1–V4)

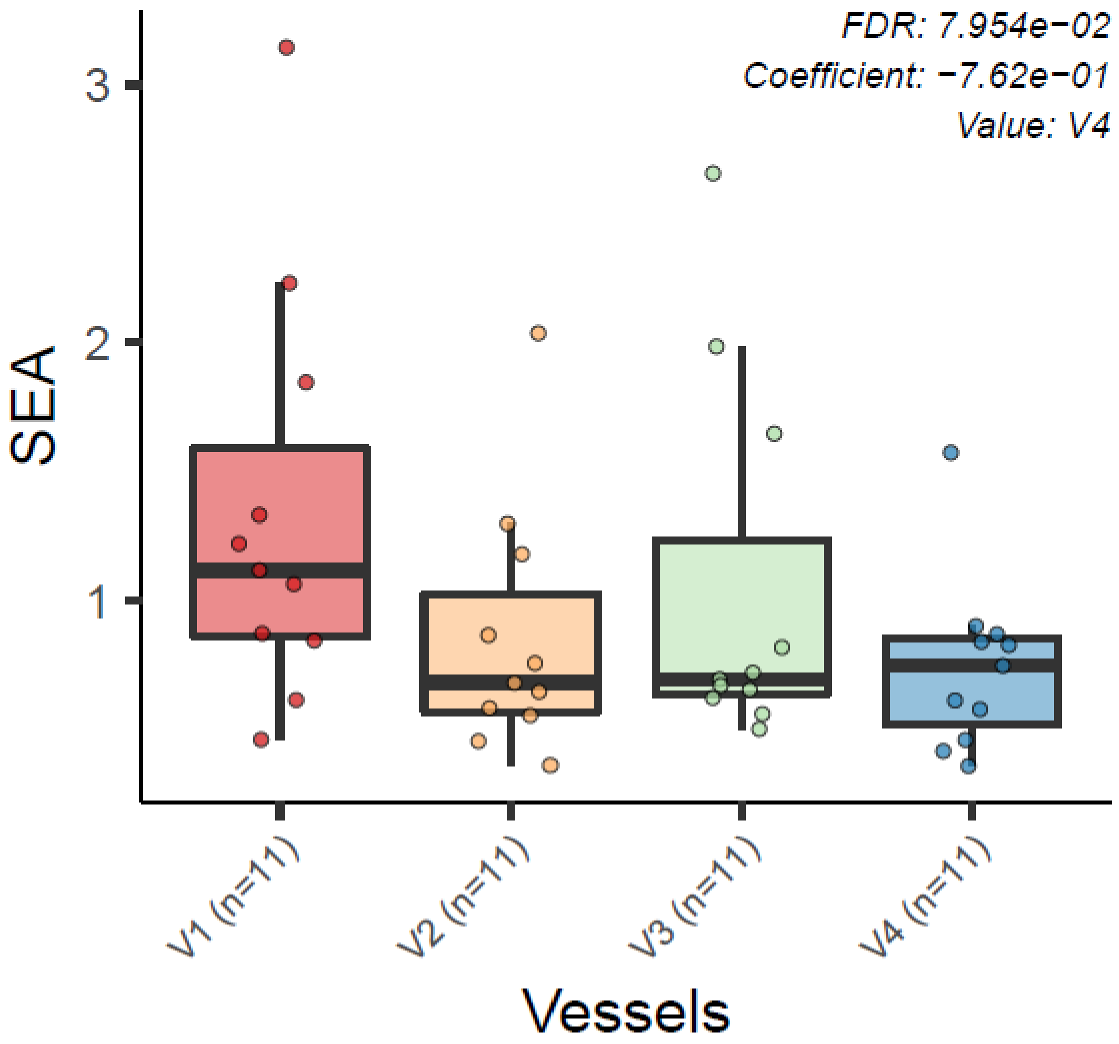

Supplementary Figure *S*2: ECCs in four vessels (V1–V4) over time (24 hours vs 48 hours)

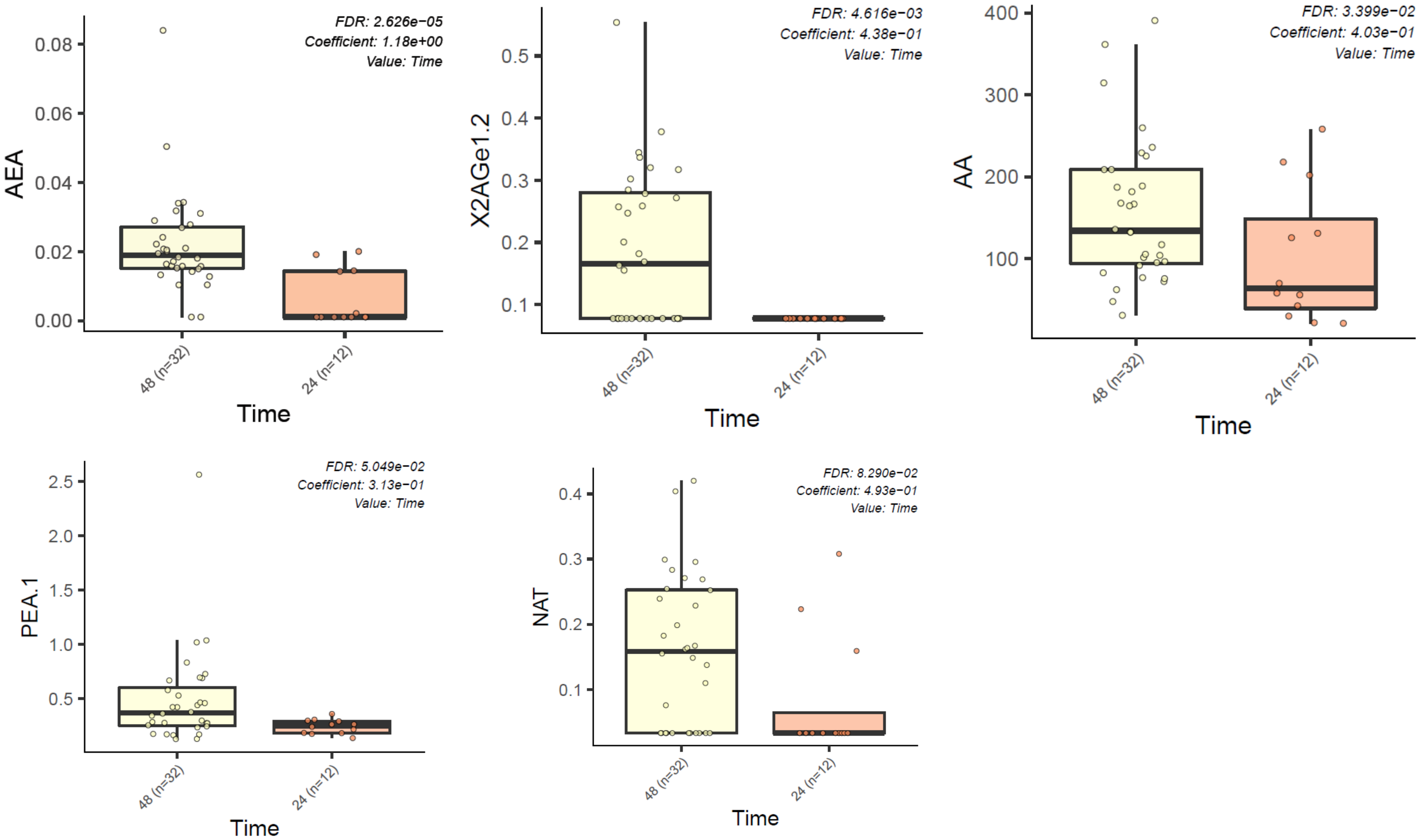

Supplement: Supplementary file 1 [file metabolites-13-00355-s001.zip › Supplementaryfiles/Supplementaryfigures.pdf]
